# *Abutilon theophrasti*’s Resilience against Allelochemical-Based Weed Management in Sustainable Agriculture – Due to Collection of Highly Advantageous Microorganisms?

**DOI:** 10.3390/plants12040700

**Published:** 2023-02-04

**Authors:** Vincenzo Tabaglio, Andrea Fiorini, Tracy M. Sterling, Margot Schulz

**Affiliations:** 1 Department of Sustainable Crop Production DI.PRO.VE.S., Section Agronomy and Plant Biotechnologies, Università Cattolica del Sacro Cuore, Via Emilia Parmense 84, 29122 Piacenza, Italy; 2 Department of Land Resources & Environmental Sciences, Montana State University, Bozeman, MT 59717, USA; 3 IMBIO Institute of Molecular Physiology and Biotechnology of Plants, University of Bonn, Karlrobert-Kreiten Str. 13, 53115 Bonn, Germany

**Keywords:** *Abutilon theophrasti*, biological control, allelopathy, fungi, bacteria, secondary metabolites, benzoxazinoids, rye mulch

## Abstract

*Abutilon theophrasti* Medik. (velvetleaf) is a problematic annual weed in field crops which has invaded many temperate parts of the world. Since the loss of crop yields can be extensive, approaches to manage the weed include not only conventional methods, but also biological methods, for instance by microorganisms releasing phytotoxins and plant-derived allelochemicals. Additionally, benzoxazinoid-rich rye mulches effective in managing common weeds like *Amaranthus retroflexus* L. have been tested for this purpose. However, recent methods for biological control are still unreliable in terms of intensity and duration. Rye mulches were also ineffective in managing velvetleaf. In this review, we present the attempts to reduce velvetleaf infestation by biological methods and discuss possible reasons for the failure. The resilience of velvetleaf may be due to the extraordinary capacity of the plant to collect, for its own survival, the most suitable microorganisms from a given farming site, genetic and epigenetic adaptations, and a high stress memory. Such properties may have developed together with other advantageous abilities during selection by humans when the plant was used as a crop. Rewilding could be responsible for improving the microbiomes of *A. theophrasti*.

## 1. Introduction

While its center of origin is uncertain, *Abutilon theophrasti* Medik. (velvetleaf) is thought to be native to China and India [1]. It was introduced across the Northern hemisphere as a fiber crop or protein food source and may have spread as a seed contaminant [1,2,3,4]. It is now a troublesome weed in major agricultural crops, such as in maize, soybeans, and cotton, and also grows in crops such as citrus, peach, and asparagus [5]. It has expanded its range to Africa, Australia, and New Zealand [6,7,8,9], and was recently identified as naturalized in Libya [10]. The species has regained interest as a fiber crop [7,11], taking up one of its old uses in Asia and for a short time in the USA, aside from its use as a medicinal plant [1,12]. In China and Kashmir, the seed is a valuable food because of its high protein content [2,8,9,13]. With the first archeological find of 7000-year-old velvetleaf seeds in a storage jar in southern Hungary, there is now evidence that velvetleaf was harvested, prepared, and stored by the Tisza culture, further supporting velvetleaf’s use as food, fiber, and medicine [14].

However, velvetleaf remains a very competitive weed. Depending on water availability, velvetleaf density, and herbicide efficacy, maize yield loss can range up to 80% [5,15,16], up to 50% in cotton [17], and up to 70% in soybean [15,18,19], causing large economic losses [2,6,20]. Competitive maize hybrids have been evaluated as a strategy to manage velvetleaf [21,22,23]; nevertheless, velvetleaf must be controlled within maize’s V3 to V8 growth stages to avoid negative effects on crop yield. Therefore, mixtures of synthetic herbicides are instead commonly recommended as pre- and post-treatments to manage velvetleaf [10,20,22]. However, herbicides can be expensive, and may persist in the environment or create selection pressures which result in the evolution of resistant biotypes. Four velvetleaf biotypes resistant to PSII herbicides have been independently reported to date [24].

Several *A. theophrasti* characteristics contribute to its competitive abilities and include its relatively large, hard-coated seeds, fast germination and emergence of the seedlings throughout the growing season, vigorous growth and large leaves, and high seed production, with long viability leading to persistent soil seed banks [2,25]. One plant can produce 8,000 seeds, with a significant percentage of them still viable for more than 50 years [2,13,25]. Moreover, velvetleaf is better colonized by arbuscular mycorrhizal fungi than other weeds, allowing velvetleaf greater access to nitrogen [26,27]. When in competition with maize, the genes of hexaploid velvetleaf involved in carbon utilization, photosynthesis, red light signaling, cell division, secondary metabolism, and phosphate availability are preferentially and more highly expressed [28]. In turn, numerous genes are downregulated in maize when grown near velvetleaf, such as those involved in photosynthesis, nutrient utilization, or signal transduction [29]. In addition, velvetleaf releases allelochemicals which are inhibitory to alfalfa, maize, and other crops (see below). In the 1980s, seed-associated endophytic microorganisms were suspected of enhancing velvetleaf´s fitness [30].

Therefore, in this article emphasizing sustainable agriculture, we shortly review results that underpin the hypothesis that *Abutilon* may organize its variable seed-coat and root-associated microbiomes with high flexibility. Additionally, this dependency on microbial diversity in site-specific soils allows for velvetleaf survival across diverse environments, for instance in those rich in benzoxazinoids and related allelochemicals.

## 2. Biological Control of *Abutilon theophrasti* by Microorganisms

Approaches to biological control of *A. theophrasti* include the use of microorganisms, plant-derived allelochemicals, mycoherbicides, or other microbial secondary metabolites. Fungi like *Verticillium dahliae*, *Alternaria abutilonis*, *Cercospora abutilonis*, *Cladosporium herbarum*, *Colletotrichum malvarum*, *Fusarium lateritium*, and others have been studied for this purpose [2,30,31,32,33,34,35,36], (Table 1). However, many of these fungi are not host specific and also infest crops (for instance, *Cercospora* leaf spot disease of sugar beet). In other cases, any biological activity damaging the weed is dependent on environmental conditions, and therefore, an unreliable tool for weed management. For instance, *F. lateritium*, a pathogenic agent of velvetleaf, reduced its growth in greenhouse experiments and in the field, but velvetleaf suppression varied from year to year, ranging from 46 to only 27% [35].

Similarly, an *Abutilon*-specific pathovar of *Colletotrichum coccodes* was genetically engineered to overexpress the oxaloacetate acetylhydrolase gene, creating a hypervirulent strain which was active at lower humidity and lethal to velvetleaf, but only through its three-leaf stage just after emergence [37]. Oxalate inhibits callose synthase by chelating the co-factor calcium, leading to less callose which weakens plant defenses [37]. Infection by other phytopathogens was also facilitated by higher oxalate levels and by exogenously applied oxalate [38,39,40,41,42]. Moreover, high oxalate amounts change the physicochemical properties of the soil, which may influence adjacent crops [43].

Another example in which pathogen efficacy for biological control of weeds was enhanced includes work which introduced the necrosis-inducing extracellular protein Nep1 from *Fusarium oxysporum.* This protein induces necrosis via ethylene synthesis and has been described as a successful biological control agent against weeds, including *A. theophrasti* [44,45]. However, genetically upgraded phytopathogens are generally not allowed anywhere in organic agriculture systems, nor in conventional agriculture in most EU countries.

Microbial secondary metabolites with phytotoxic properties against *Abutilon* produced by *Streptomyces* species [46] are able to inhibit velvetleaf germination and seedling growth by 100%. Even so, the velvetleaf seeds used in this study were surface-sterilized, disallowing any degradation or inactivation of bioherbicides by seed-surface-colonizing microorganisms, most likely reduced, if not eliminated under sterile conditions. Thus, the inhibition of velvetleaf growth found in laboratory experiments may not occur under field conditions. In other studies, hydrogen cyanide-producing *Pseudomonas putida* ATH-1RI/9 and *Acidovorax delafieldii* ATH2-2RS/1 suppress the growth of velvetleaf [47,48], though it is not known whether the bacterial-produced hydrogen cyanide was in concentrations lethal to soil organisms. It is important to note that many microorganisms can degrade hydrogen cyanide [49,50], which would remove any phytotoxic effects in the soil environment.

Overall, biological control of velvetleaf is not commonly used. Accordingly, velvetleaf is not mentioned as a target for biological control in relevant recent reviews [51,52]. As implied by Chee-Sanford et al. [53], a detailed knowledge of the entire ecosystem is a prerequisite when microorganisms will be used for the weed management. In general, it can be said that these biological control methods are still unreliable in terms of intensity and consistency, especially against weeds with gradual emergence in the field, like *A. theophrasti*.

### Natural Compounds—Monoterpenoids, Benzyl-Isothiocyanate, Phenolics, and Sorgoleone in the Management of Abutilon theophrasti

Monoterpenes can be phytotoxic when applied in high dosage [54,55]. Vaughn and Spencer tested several volatile monoterpenes from the essential oils of aromatic plants for velvetleaf control in comparison to crops like maize, wheat, alfalfa, cucumber, and soybean [56]. While seed germination of Italian ryegrass (*Lolium multiflorum* Lam.), large crabgrass, (*Digitaria sanguinalis* (L.) Scop.), and redroot pigweed (*Amaranthus retroflexus* L.) was completely inhibited by many of the essential oils, velvetleaf exhibited a tolerance similar to that found with maize and cucumber. Only fenchone, citronellol, and citral totally suppressed velvetleaf germination, but fenchone was non-selective, also inhibiting maize, wheat, and alfalfa germination. The monoterpenes also inhibited growth of maize seedlings less than cucumber and alfalfa, but greater than soybean. Consistently, most volatile monoterpenes were not specific enough to only inhibit velvetleaf germination or seedling growth.

Wolf et al. described inhibitory effects of benzyl isothiocyanate from *Carica papaya* L. seeds on velvetleaf germination and seedling growth [57]. The *C. papaya* seeds contain not only the glucosinolate glucotropaeolin but also cyanogenic glycosides; in this study, the velvetleaf seeds were surface-sterilized. The inhibition of velvetleaf by benzyl isothiocyanate was also reported by Tollsten and Bergstrom [58]. In a study of Shettle and Balke [59], effects of salicylic acid, p-hydroxybenzoic acid, caffeine, hydroquinone, and umbelliferone on shoot dry-weight accumulation of several weeds and crops were investigated in a greenhouse trial. Except for hydroquinine, velvetleaf growth was only inhibited by the compounds when exposed to high amounts (56.0 kg ha^−1^). Exudates from roots of competitive soybean cultivars have been studied for velvetleaf suppression as well; just 20 of 280 tested cultivars were able to reduce the dry weight of velvetleaf plants and did so up to 15% [60]. Dayan et al. demonstrated negative effects of sorgoleone, an allelochemical of *Sorghum bicolor* (L.) Moench on velvetleaf seedling emergence and rates of photosynthetic electron transfer in leaf discs [61]; however, the seedlings were again grown from surface-sterilized seeds. Apparently, there are presently no plant-derived compounds or extracts available which have sufficient allelopathic potential to manage velvetleaf in the field, including the benzoxazinoid-derived compounds (see below) and others [62].

Many secondary metabolites can modulate, even impoverish, species abundancies and the biodiversity of the original soil microbiome. Therefore, the use of plant and microbial allelochemicals as potential bioherbicides can lead to unexpected negative effects on valuable soil microorganisms. This consequence can be a problem when other crops relying on microbial species are subsequently cropped. Plant secondary metabolites such as glucosinolate breakdown products, p-coumaric acid, vanillic acid, salicylic acid, the flavonoids catechin and quercetin, and benzoxazinoids, gramine, and others affect microbe growth in laboratory and field studies [63,64,65,66,67,68,69,70,71,72]. Recovery of a rich biodiversity of microbial communities, for instance after fallow periods, is of utmost importance, not only for soil quality and fertility [73], but also to catalyze the complete metabolization of important plant and microbial allelochemicals such as catechol and polyphenols, including catechin and dihydroquercetin (see below) by specialized microorganisms and under special soil conditions [74].

## 3. Resilience of *Abutilon theophrasti* against Benzoxazinoid Allelochemicals from Rye (*Secale cereale* L.) Mulches

Benzoxazinoids are characteristic compounds found in maize and other gramineous species, including rye or wheat. Benzoxazinoids and phenoxazinone spontaneously produced from 2-aminophenol, a microbial degradation of benzoxazolinone, are phytotoxic [75,76]. These allelochemicals have the potential to help manage defined weed species [77,78,79]. Furthermore, rye cultivation as cover crop is increasingly gaining ground, both in conventional and, even more, in organic farming systems, due to agro-environmental benefits [80]. For instance, benzoxazinoids contained in rye mulch can manage numerous weeds such as *Chenopodium album* (L.), *Amaranthus retroflexus* (L.), *Portulaca oleracea* (L.), and others. At the same time, benzoxazinoid content in rye biomass depends on cultivar and growth stage [81,82,83]. In this respect, the termination timing of rye cover crop may influence the allelochemical concentrations in rye mulches which decrease with plant age [83], but, if delayed, also allow more biomass production and a concomitant physical control of weed seedlings emergence [84].

**Figure 1:**
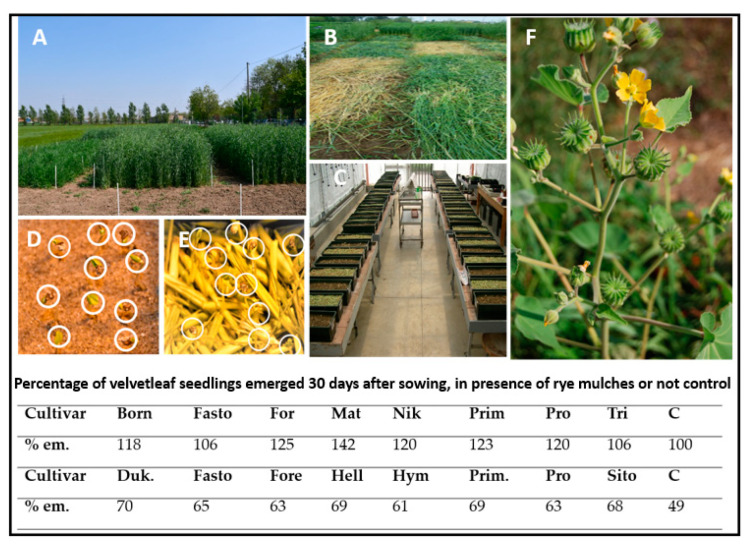
(**A**,**B**): Preparation of rye mulches at the Università Cattolica del Sacro Cuore research station in Piacenza, Italy. (**C**): Rye mulch covered and control planter boxes for velvetleaf germination experiments in the greenhouse. (**D**,**E**): Emergence of velvetleaf seedlings (white circles) in pots with rye mulch (**E**), only seedlings overtopping the rye mulch cover are circled) in comparison to the control (**D**). (**F**): Flowering velvetleaf plant in a maize field. Table—Abbreviations: % em: percentage of emerged velvetleaf seedlings; cultivars: For, Forestier; Mat, Matador; Nik, Nikita, Prim, Primizia; Pro, Protector; Tri, Treviso; Duk, Dukato; Fore, Forestal; Hell, Hellvus; Hym, Hymonta; Sito, Sito70; C: control.

Therefore, several approaches have been undertaken to manage *A. theophrasti* using rye mulch. Mulches were prepared from different rye cultivars differing in their benzoxazinoid content, ranging from about 200 to 550 μg g^−1^, and were used for pot experiments [83,85] (Figure 1). The mulches effectively reduced seedling growth of three weeds (*C. album*, *A. retroflexus*, *P. oleracea*); however, none of the mulches inhibited the growth of velvetleaf seedlings (Figure 1). Interestingly, the number of emerged velvetleaf seedlings in the mulched pots was 35% higher than in the control pots on average, though not statistically different. These results corroborate previous outcomes in other pot and field studies [82,85]. The failure to manage velvetleaf with rye mulches and velvetleaf’s subsequent success in maize fields point to efficient strategies of the plant to cope with benzoxazinoid allelochemicals.

### 3.1. Microorganisms Associated with Abutilon theophrasti and Microbial Role in Benzoxazinoid Tolerance

A number of fungal species are known to colonize the seed coat of *A. theophrasti*, including *Cordyceps sinensis*, *Cephaliophora tropica*, *Mandurella mycetomatis*, *Actinomucor elegans*, *Aspergillus flavus*, *Trichoderma viridae*, *Penicillium* spp., *Fusarium* spp., *Alternaria alternata*, and *Verticillium* spp. [30,31,53], while *Cladosporium cladosporioides* and *Epicoccum purpurascens* are seed-borne fungi (Table 2). Bacteria isolated from the seed surface were identified as species belonging to the genera *Bacillus*, *Flavobacterium*, and *Pseudomonas*. More than half of the seeds contained seed-borne bacteria belonging to the genera *Pseudomonas*, *Alcaligenes*, and *Flavobacteria* [30]. There is also evidence for site-specific composition of seed-colonizing microorganisms. For example, velvetleaf seeds collected from three sites (Meleti, Pralboino, and Gambara) in Northern Italy were tested to verify the presence of endophytic fungi [86]. Seeds were surface-sterilized and then incubated at 25 °C for germination. *Alternaria alternata* was identified in 27% of the radicles and *Fusarium* spp. was identified in 36% of the radicles from seeds collected at Meleti site. In 42% of radicles from seeds collected at the Pralboino site, *Fusarium* species were present. Seeds from the Gambara site led to seedlings without these fungal endophytes. The isolation of fungi from 6-day-old radicles suggests migration from the seed to the root system. *Alternaria alternata* and *Fusarium* species, frequently associated with *A. theophrasti* seeds, are often endophytic [87,88].

*Actinomucor elegans* was found on velvetleaf seeds purchased from Herbiseed (Twyford, UK), which were harvested from the Herbiseed farm Gradiste, Serbia, in 2011 and 2012. *Actinomucor elegans* was not found on seeds harvested in 2013 from another Herbiseed farm site. These findings support the assumption that the habitat of the parent plant is crucial for the variable microbial species composition associated with *A. theophrasti* seeds. It is known that root microbiomes contain microorganisms attracted from the soil microbial community [89]. *Actinomucor elegans*, re-isolated from velvetleaf roots, was identified as a “consortium”, harboring numerous bacteria, for instance, *Pantoea ananatis* and *Stenotrophomonas maltophilia* and the yeast *Papiliotrema baii*. This consortium has a larger metabolic potential than its isolated members [90].

**Figure 2:**
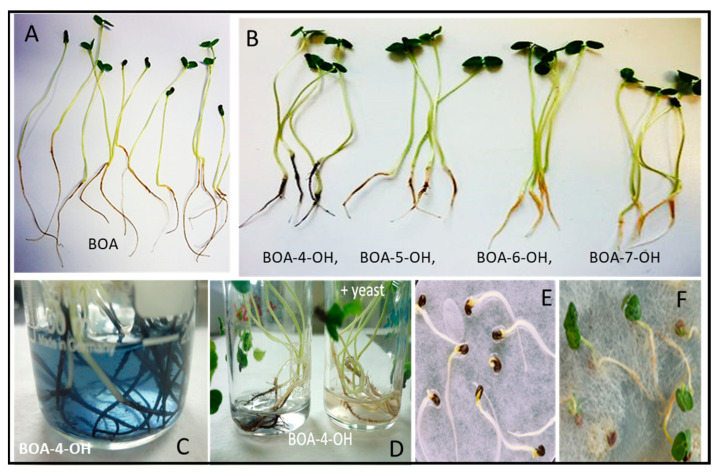
(**A**): Seedlings after 24 h BOA incubation, leaves are not impaired. (**B**): Incubations of seedlings with the four BOA-OH isomers. Seedlings are shown after the incubation: Only BOA-4-OH led to roots covered with a bluish-black polymer. (**C**): Roots exposed to BOA-4-OH during incubation. (**D**): Addition of the yeast *Papiliotrema baii*, a member of the *Actinomucor elegans* consortium, prevents polymer covers on the root surfaces. (**E**): *A. elegans* consortium has no damaging effects on germinating velvetleaf. (**F**): Healthy velvetleaf seedlings are cocooned by *A. elegans* hyphae.

Incubations of the *A. elegans* consortium with benzoxazolinone (BOA), the first degradation product of benzoxazinone, did not inhibit fungal growth or lead to any degradation or detoxification products within one week. Additionally, phenoxazinone, a phytotoxin derived from 2-aminophenol, a microbial degradation product of BOA, did not impair fungal growth. Even under nutrient deficiencies, the consortium did not use these compounds as a C or N source [91,92]. Nevertheless, the consortium responded to high BOA concentrations by enhancing the glucosyl ceramide content of membranes isolated from the consortium [93]. The consortium released tryptophan as the most prominent compound into the medium. Tryptophan release increased in the presence of BOA during the first week of incubation but dropped below the control level with increasing incubation times over four weeks [92]. It is doubtful that the released tryptophan is used for auxin production by the Trp-dependent IAA biosynthetic pathway when the consortium colonizes velvetleaf roots, since its root exudates have the potential to reduce IAA production significantly. This possibility is important since high IAA amounts are deleterious to velvetleaf growth [94]. The consortium had no inhibitory effect of germination or seedling growth (Figure 2). In contrast, *A. elegans* hyphae seems to protect the seedlings ([90,95], and see below).

Isolated *P. ananatis* and the entire consortium, but not the isolated yeast, are able to nitrate the plant-produced detoxification intermediate BOA-6-OH, resulting in 6-hydroxy-5-nitrobenzo[*d*]oxazol-2(3*H*)-one (NO2-BOA-6-OH). In contrast, *P. baii* formed only a dimer of BOA-6-OH [90,96]. NO2-BOA-6-OH was produced by velvetleaf root surface proteins and was also identified in the incubation media of velvetleaf when supplemented with BOA-6-OH. This nitroaromatic compound in concentrations of 8 to 86 µM enhanced *A. theophrasti* root growth at pH 5 but had no effect on shoot growth. The I_50_ value was 300 µM for root growth at pH 5 and 700 µM for shoot growth at pH range of 4 to 6. Velvetleaf root surface proteins that included microbial sources were able to reduce NO2-BOA-6-OH up to 90% when assayed in vitro with the compound. In contrast, *Lepidium sativum* (L.) root and shoot growth was inhibited by 50% and 90%, respectively, with 86 µM NO2-BOA-6-OH [96]. NO2-BOA-6-OH? I_50_ values were 20 μM at pH 6.0 for *L. sativum* root growth and 10 μM for its shoot growth [96]. Thus, NO2-BOA-6-OH can be deleterious for other plants, but apparently not for velvetleaf grown from non-sterilized seeds.

### 3.2. Defense Strategies against Benzoxazolinone (BOA)—Uptake, Detoxification, Membrane Repair, and Polymerizations at the Root Surface

Velvetleaf detoxifies benzoxazolinone (BOA) differently than other important summer weeds, such as *Chenopodium album*, *Amaranthus retroflexus*, and *Portulaca oleracea*, but also many others. While BOA uptake by velvetleaf roots was similar to uptake by *C. album*, *A. retroflexus* and *P. oleracea*, velvetleaf roots produced remarkably fewer detoxification products when 20 to 40 µmol BOA was applied. Additionally, less BOA was transported to the shoots where almost no detoxification products were found in velvetleaf shoots, in contrast to the above-mentioned weed species [97].

Velvetleaf seedlings not colonized by the *A. elegans* consortium exhibited a different detoxification behavior by accumulating higher amounts of BOA-6-O-glucoside in roots and shoots during incubation with 500 µM BOA [90,97]. When colonized by the consortium, more BOA-5-O-glucoside than BOA-6-O-glucoside accumulated in velvetleaf roots, suggesting that the consortium influences the position of BOA hydroxylation. Without the fungus, velvetleaf seedling length was affected by BOA-6-OH and BOA-5-OH in comparison to the control, while BOA-6-OH led to a higher inhibition than BOA-5-OH. Velvetleaf seedlings grown from seeds not associated with *A. elegans* from the Meleti field also had more BOA-6-O-glucoside than BOA-5-O-glucoside.

The responsible glucosyltransferase activity could be extracted from plant cell wall material whereas the cytosolic fraction was inactive. The plant-produced intermediates BOA-6-OH and BOA-5-OH, as well as the synthetic isomers BOA-4- and -7-OH, led to brownish and black roots but otherwise had no effect on the phenotype, although all these compounds also induced H_2_O_2_ production, indicated by high catalase activity at the root surfaces [90] (Figure 2). High catalase activity was found in the bacterial isolates and a yeast belonging to the *A. elegans* consortium (see above). On root surfaces, peroxidase, laccase, and phenol oxidase activities could also be measured, so at least a portion of these activities are from root-colonizing microorganisms [90]. Acting in concert, the activities are responsible for BOA-OH polymer formation at the root surface (Figure 2). It is unknown whether these polymers permanently cover roots under natural conditions, because microorganisms, for instance the yeast *P. baii*, associated with the consortium, remove BOA-4-OH over time, also preventing its polymerization.

Incubation of velvetleaf seedlings with BOA-6-OH, which has a higher toxicity than BOA, damages membrane lipids and fatty acids in the root tips. Decreased content of α-linoleic acid (18:2), phosphatidylinositol, and phosphatidylcholine were measured in root tips within 30 min of incubation. However, membrane repair was as rapid as found with maize root tips and control levels were reestablished within one hour after exposure to the compound [98]. Thus, even when BOA-6-OH would accumulate in velvetleaf root tips, which had not yet been observed, the plant has powerful mechanisms for rapid membrane repair.

Like many other natural compounds, benzoxazinoids are considered templates for the design of new herbicides. Substitutions such as hydroxylation, especially in position 6, activate BOA, allowing further substitutions, for instance, in the case of 6-hydroxylation, in which nitration in position 5 reduces the phytotoxicity for *Abutilon*.

## 4. Secondary Metabolites of Velvetleaf

**Figure 3:**
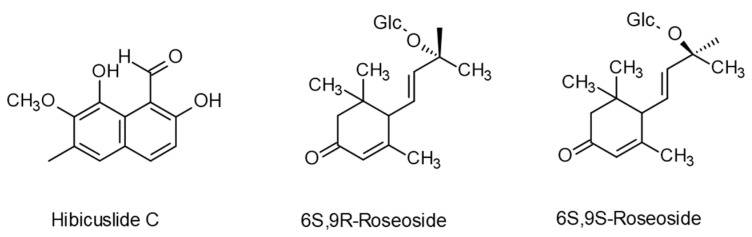
Less known secondary metabolites found in extracts of *A. theophrasti:* hibiscuslide C and roseosides, identified by Hwang et al. and Mamadalieva et al. [106,107].

The allelopathic activity of aqueous and methanolic extracts from all parts of *A. theophrasti* has often been studied. In particular, exudates of the glandular trichomes of *Abutilon* are phytotoxic [18,99]. Inhibitory effects of aqueous extracts from velvetleaf plants on maize, tomato, sugar beet, oilseed rape, oats, cucumber, radish, soybean, and sunflower have also been described [100,101,102]. Paszkowski and Kremer [103] identified the flavonoids delphinidin, cyanidin, quercetin, myricetin, (+), (–)- catechin, and -epicatechin in extracts from velvetleaf seed coats. Kaempferol 3-O-β-(6″-p-coumaroyl) glucopyranoside was identified in methanolic extracts of above-ground velvetleaf tissue [104]. Tian et al. [105] reported gallic acid, protocatechuic acid, tannin, catechin, vanillic acid, caffeic acid, ferulic acid, rutin, and quercetin in extracts from the root, stem, leaves, seeds, and exocarp. Hibiscuslide C isolated from velvetleaf above-ground tissue had antifungal activity in *Candida albicans* [106]. In addition, Mamadalieva et al. [107] isolated (6S,9R)-roseoside and (6S,9S)-roseoside from the aerial parts of the plant (Figure 3).

**Figure 4:**
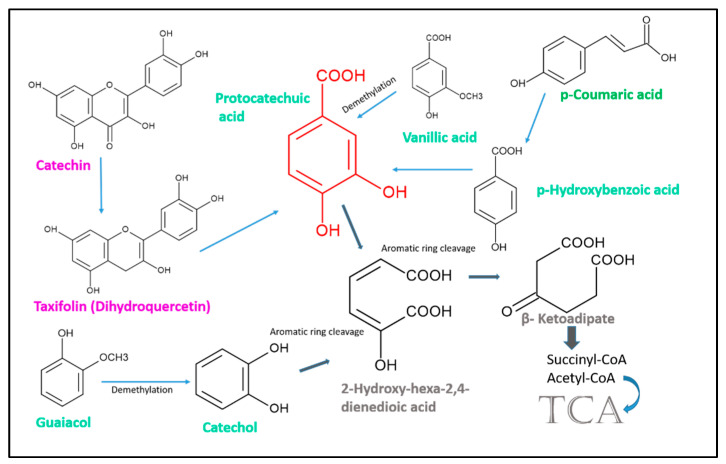
Some steps of the microbial degradation of catechin and taxifolin as members of the flavonoids (magenta), p-coumaric acid, a cinnamic acid derivative (green), and several simple phenolic compounds (petrol). Many phenolic compounds are degraded via the central intermediate protocatechuic acid, and others via catechol. Catechol and protocatechuic acid (red structure) undergo either ortho- or meta- cleavage of the aromatic ring, resulting in 2-hydroxy-hexa-2,4-dienedioic acid and β-ketoadipate. These compounds are converted to succinyl-CoA and acetyl-CoA for entering the citrate cycle (TCA).

Aqueous extracts of velvetleaf seed coats can inhibit multiple soil fungi. Single flavonoids from velvetleaf leaves and stems had negative effects on fungi with seed-decomposing properties [104]. It is possible that not all bioactive secondary metabolites of velvetleaf are yet identified, but almost all of those known are widespread across plants and can act as allelochemicals. Additionally, the more recently identified roseosides are not specific to velvetleaf. They have been found in *Adina* and a *Polygonum* species and in *Centella erecta* [108,109,110]. Except for the roseosides and hibiscuslide C, all the identified phenolic compounds are known for complete degradation by bacteria (Figure 4).

Consistently, these compounds in their monomeric form do not accumulate appreciably in the soil, but perhaps do so within the rhizosphere in amounts that are sufficient for allelopathic inhibitions or other functions. More likely, the compounds in their specific combination and amount may have defined chemotactic properties, attracting microorganisms valuable for velvetleaf fitness at a given growing site. Additionally, degradation intermediates, when sufficiently stable, may have defined functions, as was shown for protocatechuic acid derived from catechin [69]. Protocatechuic acid is phytotoxic, but the compound also has antioxidant activity [111]. Microbial conversions and degradation products of the parental phenolic secondary metabolites increase the number of bioactive derivatives dramatically, at least temporarily, but their roles, particularly short-term ones, in the plant–microbe interaction are understudied. Is the production of common and unusual microbial conversion products one reason for the deleterious impacts on other plants, although these plants synthesize the same compounds and exude them? Presently, this question cannot be answered. Since many phenolic compounds have antioxidant properties, they may also protect beneficial, root-colonizing microorganisms against oxidative stress when attacked by pathogens or when faced with antibiotics produced by competitive species used for allelopathic activity. When highly concentrated, the protection provided by phenolic compounds with antioxidant properties will reverse and become toxic.

## 5. Considerations and Further Perspective

This short review demonstrates the extraordinary capability of *A. theophrasti* to withstand attempts for its biological control, for instance by allelochemicals. Velvetleaf seems equipped with a combination of highly efficient, unique strategies, in addition to those mentioned in the introduction, for high tolerance against allelochemicals such as benzoxazinoids. One of these strategies is certainly featured by attracting the most suitable soil microorganisms, thus avoiding competitive conflicts among the attracted species and the already established species. This strategy allows enormous flexibility and an optimal adaptation to different growing sites with specific pedo-climatic conditions and interspecific interactions between micro- and macro-biota. The mode of attraction is unclear, but the exuded secondary metabolites, perhaps specific mixtures of them, are certainly involved. The microbe-*Abutilon* interactions may be of crucial importance for the success of the plant.

Another strategy encompasses the translocation of allelochemical detoxification and elimination into the cell wall compartment and to the root surface, where microorganisms take over the work. This strategy seems to be highly developed in velvetleaf and presents a distinctive difference to many other weeds and crops. The assumption is in line with the finding that bentazon metabolites were not detected in velvetleaf cells prepared from calli produced in axenic culture, in the velvetleaf hypocotyl sections, or in the media used for incubations. In contrast, soybean cells from suspension cultures detoxified bentazon to 6-*O*-ß-glucose-bentazon and 8-*O*-ß-glucose-bentazon [112]. It can also be hypothesized that the plant has evolved mechanisms for suppressing the release of phytotoxins produced by microorganisms that intrude the rhizosphere. The inhibition of bacterial IAA production points in this direction.

Other reasons for *Abutilon’s* fitness may be related to its old use as a crop, where plants with desired new properties were selected by humans for further cultivation [1], while escapes of the cultured plant to the wild might allow velvetleaf more opportunity to select suitable microorganisms from its soil. During rewilding, domesticated *Abutilon* could reestablish or even improve its microbiomes for re-attaining biodiversity levels previously lost or diminished during domestication. Diminished microbiome diversity as a result of cultivation is a known phenomenon for some crops [113,114], and rewilding to improve microbiomes is being explored to strengthen modern crops [115], if such restoration is still possible given the loss of microbial biodiversity due to human activities [116]. When experiencing stress conditions during rewilding, *Abutilon* might have also evolved a strong transgenerational stress memory that includes epigenetics, overaccumulation of proteins involved in managing stress, in dynamic proteome modulation and rapid protein interactions to adapt quickly to stress conditions [117], while concurrently maintaining high seed production. Presently speculative, *Abutilon* may collect preferentially microorganisms with a well-developed stress memory [118], although the molecular mechanisms of selective capture are enigmatic.

Ghanizadeh and James studied the establishment of velvetleaf in different regions of New Zealand and found that the invasion dynamics depend on climate niches with different local temperatures and daily solar radiation [9]. For this reason, velvetleaf is problematic only in one region in the North Island where temperatures are higher, suggesting that there are limiting factors for velvetleaf and its ability to adapt [9], although the influence of soil microbiomes was not included in the study. It is certainly worthwhile to study the genetic, biochemical, and physiological traits of this weed more intensively to disclose vulnerable features and strong attributes important for survival. In this sense, *A. theophrasti* could be a role model for the development of new strategies and environmentally acceptable technologies that strengthen crops when in competition with weeds.

## Figures and Tables

**Table 1:** Microorganisms with biocidal effects on velvetleaf.

Microorganisms	Mode of Action	Note	Reference
**Fungi**			
*Alternaria abutilonis*	leaf spot		[2]
*Cercospora abutilonis* *Cerospora* spec. complex?	Leaf spot	species needs additional identification	[2]
*Cercospora althaeina* Sac.	Leaf rot		[2]
*Cladosporium herbarum*	secondary leaf spot		[2]
*Colletotrichum coccodes*	foliar pathogen, plant death; suppression of plant defenses	enhancement of virulence by mannose and oxalic acid, NEP1 for hypervirulent transgenic strain	[2,39,40,45]
*Colletotrichum malvarum*	leaf spot		[2]
*Fusarium lateritium*	growth suppression		[2,35]
*Fusarium oxysporum*	24-kDa protein Nep1 induces necrosis		[44]
*Macrophomina phaseoli*	stem rot		[2]
*Phyllosticta althaeina* Sacc.	Leaf rot		[2]
*Phymatotrichum omnivorum*	root rot		[2]
*Puccinia heterospora*	rust	possible mycoherbicide	[2]
*Verticillium dahliae, V. nigrescens*	wilt	suggested as a biocontrol agent, unspecific	[2]
**Bacteria**			
*Acidovorax delafieldii* ATH2-2RS/1	inhibits growth		[48]
*Pseudomonas putida* ATH-1RI/9	inhibits growth		[48]
*Pseudomonas* spec isolates Pp001 and Pf239	Reduction of seedlings emergence weaken seedlings		[32]
*Streptomyces isolates*	inhibits germination, growth		[46]

**Table 2:** Microorganisms known to be associated with velvetleaf. Locations: 1 Missouri Agronomy Research Center, 2 Osage County, MO, USA; 3 Meleti, 4 Gambara, 5 Pralboino, Italy; 6 Gradiste, Serbia; 7 ASSET agricultural fields IL, USA; 8 USSEU undisturbed site IL, USA.

Microorganisms	Seed Surface	Within Seeds	Location	Activity	Reference
**Fungi**					
*Actinomucor elegans,* strain AF157119	x x		6 7	protects *Abutilon* seedlings	[53,89,90,91]
*Alternaria alternata*	x	x	1, 2, 3		[30,86]
*Aspergillus flavus*	x		7		[53]
*Cephaliophora tropica* strains	x		8		[53]
*Cladosporium cladosporioides*	x		1, 2		[30]
*Cordyceps sinensis*	x		7, 8		[53]
*Epicoccumpurpurascens*	x		1, 2	inhibits seed-borne bacteria	[30]
*Fusarium* spp.	x	x	1, 2, 3, 4, 5	moderate antagonism toward test fungi	[30,86]
*Madurella mycetomatis*	x		8		[53]
**Bacteria**					
*Alcaligenes* spec. isolates			1, 2	highly antagonistic to seed fungi	[30]
*Bacillus* spec. isolates			1, 2		[30]
*Bacillus subtilis*			1, 2		[30]
*Flavobacterium* spec.			1, 2		[30]
*Pseudomonas* spec. isolates			1, 2		[30]

## Data Availability

Not applicable.
